# Two-Stage Sternotomy Approach to Hostile Chest Reentry for Orthotopic Heart Transplantation

**DOI:** 10.3390/jcm14041251

**Published:** 2025-02-14

**Authors:** Alden J. Dunham, Leonardo Paim N. da Costa, Lucian Lozonschi

**Affiliations:** 1 Morsani College of Medicine, University of South Florida, Tampa, FL 33602, USA; 2 Division of Cardiothoracic Surgery, University of South Florida, Tampa, FL 33602, USA; ldacosta@usf.edu (L.P.N.d.C.); lozonschi@usf.edu (L.L.)

**Keywords:** complex sternotomy, heart transplantation, heart failure

## Abstract

**Background:** Repeat cardiac operations carry increased risk of morbidity and mortality due to adhesions between the heart and adjacent structures, which increase the complexity of redo sternotomy and the potential for reentry injury. Preoperative CT imaging has been associated with a decreased risk for reentry injury. **Methods:** We present a series of two cases of complex thorax reentry in which a stepwise sternotomy approach enabled safe reentry. **Results:** Both patients tolerated the procedure well, with successful orthotopic heart transplantation in each case. A thorough review of the preoperative CT imaging enabled surgical planning to safely navigate structures at high risk for reentry injury. **Conclusions:** This series demonstrates the utility of two-stage sternotomy in complex thorax reentry cases and the importance of preoperative imaging for the safe surgical planning of such an approach.

## 1. Introduction

Repeat cardiac surgery carries increased risk for morbidity and mortality compared with the initial operation [1,2]. Redo sternotomies are more technically complex, and adhesions and anatomic alterations that result from prior surgeries create the possibility for reentry injuries. In the existing literature, the incidence of reentry injury varies, but the consequences are consistently severe with significant increases in perioperative morbidity and mortality [1,2,3]. Hamid et al. reported a low rate of reentry injury at 2.7% but noted a perioperative mortality of 26% in patients who suffered such an injury compared with 9% in patients who did not. Park et al. described a higher frequency of reentry injury at 9.0% while still noting elevated hospital mortality at 18.6% vs. 6.5% in those without reentry injury. Risk factors for reentry injury include an increased number of prior cardiac surgeries and a history of chest radiation. Preoperative CT imaging is associated with a decreased incidence of reentry injury as structures at risk for damage can be identified, and strategies to mitigate those risks can be enacted [1,2,3,4].

One strategy to mitigate the risk associated with complex chest reentry is deep hypothermic circulatory arrest (DHCA), when one foresees that the heart or the aorta are in intimal contact with the sternum. While this approach offers protection against hemorrhage and ischemia in the case of otherwise catastrophic reentry injury, it carries serious risks of its own. Patients who undergo DHCA for longer than 40 min demonstrate increased rates of brain injury [4,5]. Deep hypothermia is additionally associated with coagulopathy through delayed clotting factor and platelet function, and end-organ hypoperfusion results in increased rates of renal failure, hepatic injury, metabolic disturbances, and systemic inflammatory response syndrome [6,7,8,9,10].

The use of a partial sternotomy to enable a stepwise approach to complex reentry is unreported in the existing literature. While partial sternotomies have been described to minimize the invasiveness of certain amenable procedures such as aortic root replacement, aortic valve replacement, and mitral valve repair, the utility of a partial sternotomy to establish cardiopulmonary bypass (CPB) prior to completing a high-risk sternotomy has not been discussed [11,12,13]. In this case series, we present two cases of complex redo sternotomy in which a thorough analysis of preoperative imaging informed a two-stage sternotomy approach to safe chest reentry without the need for deep hypothermic circulatory arrest.

## 2. Detailed Case Descriptions

### 2.1. Case 1

A 61-year-old male with HFrEF secondary to ischemic cardiomyopathy s/p Heartmate III left ventricular assist device (LVAD) and automatic implantable cardiac defibrillator (AICD) implantation three years prior presented to the clinic for consideration of orthotopic heart transplantation. The patient had a history of a bicuspid aortic valve for which he underwent surgical aortic valve replacement (SAVR) with a bioprosthetic valve 17 years prior and redo SAVR with a mechanical valve nine years prior. He had been turned away from multiple outside transplant centers due to concerns about the safety of transplantation given his history of three prior sternotomies and his stability on LVAD. However, the patient remained interested in a transplant and was accepted for a transplant evaluation workup at our center listed as status 4. The patient acutely presented to the hospital due to a painful AICD shock delivered overnight. In the hospital, the patient was upgraded to status 2e, and his acute decompensated heart failure was managed medically while awaiting transplantation.

**Figure 1:**
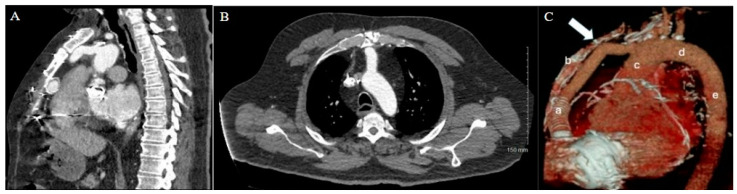
Patient 1 CT chest. (**A**) Sagittal view and (**B**) axial view reveal extensive adhesions between LVAD outflow graft and superior retrosternum. (**C**) 3D reconstruction with labeled structures LVAD outflow graft (a), sternum (b), ascending aorta (c), aortic arch (d), and descending aorta (e) highlighting the point of maximal LVAD graft–sternal contact (white arrow).

**Figure 2:**
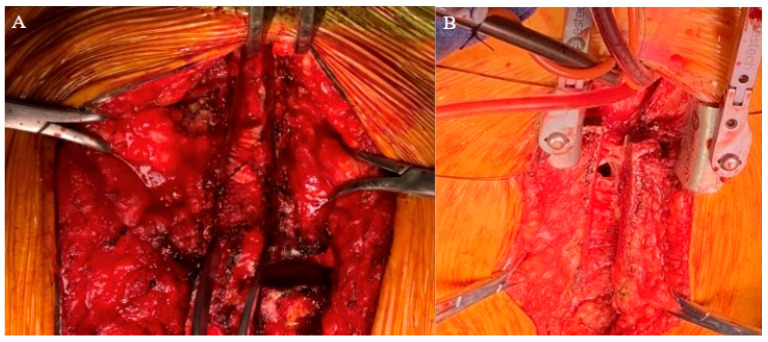
Intraoperative images of (**A**) Patient 1 and (**B**) Patient 2 highlight dense adhesions with two-stage sternotomy approach.

As expected, the patient’s extensive history of cardiac surgery with three prior sternotomies made for a complicated reentry to the thorax. A review of the preoperative chest CT demonstrated tight adhesions between the LVAD outflow graft and the superior retrosternal surface (Figure 1). The precise anatomic level of these adhesions and the safe portion of the sternum without adhesions was localized by leveraging the sternotomy wires as landmarks. Intraoperatively, a partial sternotomy was made from the xiphoid process, inferiorly, until the third intercostal space (Figure 2A). At this point, the outflow graft was mobilized, and aortic cannulation was established via the LVAD outflow graft, and IVC drainage was established via percutaneous peripheral femoral vein access. The inferior portion of the sternum was then carefully dissected away from the heart, mediastinum, and LVAD outflow graft. Due to dense adhesions between the LVAD outflow graft and the sternum, a section of periosteum was left attached to the graft.

**Figure 3:**
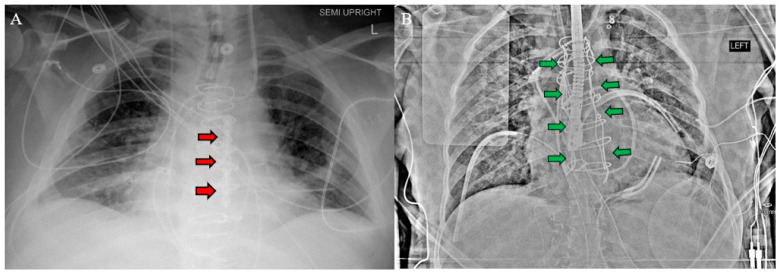
Postoperative chest X-ray for (**A**) Patient 1 and (**B**) Patient 2 demonstrate sternal plate used in Patient 1 (red arrows **A**) and Robicsek technique used in Patient 2 (green arrows **B**).

After establishing cardiopulmonary bypass through the inferior partial sternotomy, the two-stage sternotomy was then completed with the separation of the superior sternum with the oscillating saw. Direct SVC cannulation was established, and the remainder of the heart and great vessels were dissected. Severe, densely calcified adhesions were encountered between the aorta and the pulmonary artery, which required extensive dissection. Following this complex dissection, cardiectomy and transplant were performed routinely. After transplantation, the chest was left open and packed for hemostasis, and the patient was returned to the OR on POD 1 for sternotomy closure. One longitudinal sternal plate was used with sternal wires posterior to the plate to close the sternum (Figure 3A).

The postoperative course was complicated by prolonged inotrope requirement with difficulty weaning off dobutamine, substernal hematoma requiring drainage, and left internal jugular vein occlusive DVT requiring mechanical thrombectomy. These complications were all expected given the patient’s medical condition and unrelated to the two-stage sternotomy. Successful function of the transplanted heart was demonstrated with normal cardiac output on RHC, LVEF of 55–60% on TTE, and absence of evidence of acute rejection on endomyocardial biopsy (EMB). The patient was discharged 31 days post-transplant. As of six months post-transplant, the patient remained clinically stable and demonstrated excellent graft function and no evidence of rejection.

### 2.2. Case 2

A 26-year-old female presented to the transplant clinic for consideration of combined OHT and OLT. She had undergone OHT as an infant due to idiopathic cardiomyopathy and subsequent CABG nine years prior to presentation to treat cardiac allograft vasculopathy. The medical history additionally included severe tricuspid regurgitation, cirrhosis secondary to congestive hepatopathy, post-transplant lymphoproliferative disorder with conversion to diffuse large B cell lymphoma, and stage 3 chronic kidney disease. She was approved for OLT and OHT pending pre-transplantation dental impaction when she presented to the hospital with an acute dental infection. She underwent dental extraction and was optimized medically for transplant.

**Figure 4:**
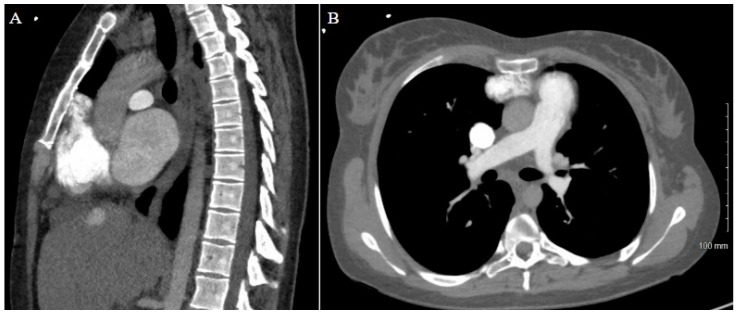
Patient 2 CT chest w/contrast. (**A**) Sagittal view and (**B**) axial view reveal extensive adhesions between right atrium and inferior retrosternum.

The patient’s history of repeated sternotomies again created a challenging reentry to the thorax. The preoperative CT revealed extensive adhesions between the right atrium and the inferior retrosternal surface with no pericardium present (Figure 4). Due to multiple previous interventions, the patient did not possess adequate peripheral vessels amenable to cannulation. The two-stage sternotomy approach was again utilized to address these adhesions. A partial sternotomy was initiated at the sternal notch and proceeded inferiorly to the third intercostal space, where an inverted T incision was made (Figure 2B). After the partial sternotomy, the aorta and SVC were carefully dissected and directly cannulated, and CPB was initiated. After CPB was initiated, the stepwise sternotomy was completed with the separation of the remaining inferior portion of the sternum. Due to the dense adhesions between the inferior sternum and the right atrium, the atrium was penetrated by the bone saw and the atrial cavity was directly exposed on opening of the chest. Fortunately, the CPB made for a bloodless field, and direct repair of the right atrial wall with a running Prolene suture was easily achieved. The remainder of the heart was dissected, with cardiectomy and transplantation performed according to standard procedure. The sternotomy was left open until the patient underwent OLT on POD1.

Prior to OLT on POD1, the CVP was mildly elevated, so continuous renal replacement therapy was initiated to remove fluid. The CVP came down appropriately, and OLT was performed without complication. The chest and abdomen were both simultaneously closed after liver transplantation, with a Robiscek technique utilized for sternal closure (Figure 3B). The patient recovered in the CTICU until POD17 as she required a prolonged course of dobutamine inotropic support, which was anticipated. The postoperative evaluation revealed a significant improvement in LVEF, tricuspid regurgitation, and cardiac output with no evidence of rejection on EMB. The patient remained in the CTICU until transfer to the general transplant medicine floor on POD17 and was discharged on POD58. As of three months post-transplant, the patient remained clinically stable with excellent graft function and no evidence of rejection.

## 3. Discussion

This case series highlights two critical points. The first is the utility of a partial sternotomy to enable a stepwise, two-stage approach to complex chest reentry. The second is the importance of a thorough imaging review as part of preoperative planning for such an approach. In both cases, stepwise sternotomy enabled the establishment of CPB prior to operating on sites that are at high risk of injury. In our first case, an initial inferior sternotomy established sufficient access for IVC drainage and aortic cannulation via the LVAD outflow graft. Similarly, in the second case, an initial superior sternotomy enabled access to direct aortic and SVC cannulation. As a result, predictable reentry injury to the right atrium was of little consequence as the patient was already on CPB, and catastrophic hemorrhage was avoided. This approach is in contrast to the exiting literature wherein reentry injury is infrequent but catastrophic with significantly elevated hospital mortality rates.

A careful review of the CT imaging was crucial to the preoperative planning of both cases of stepwise sternotomy. The preoperative imaging identified not only structures at risk of reentry injury but also the specific site at which this risk was present. By utilizing landmarks identified in the imaging, we were able to formulate an individualized two-stage approach to reentry that was safe for each patient. With one patient demonstrating inferior retrosternal adhesions and one demonstrating superior retrosternal adhesions, understanding this patient-specific anatomy was necessary to strategize a safe approach to stepwise sternotomy.

The utilization of two-stage sternotomy to establish CPB and mitigate the risk of reentry injury enabled us to avoid the necessity for other protective strategies including DHCA. By maintaining normal end-organ perfusion throughout the procedure, we avoided the neurologic, renal, and hepatic injury associated with hypoperfusion in DHCA. Similarly, by averting the need for deep hypothermia, we avoided the associated coagulopathy. By avoiding prolonged rewarming time and minimizing blood loss, we expect shorter operative times, fewer transfusion requirements, and shorter overall recovery for patients. We expect that these benefits of this approach will apply across cardiothoracic surgical procedures. Any patient with a hostile thoracic anatomy, including those with anterior chest wall deformities rather than prior sternotomies, who require cardiac surgery could theoretically benefit from this technique. The conceptual basis of this approach is built on typical cardiothoracic operative techniques with an emphasis on detailed preoperative imaging review, and, thus, the technique should be readily accessible.

Both patients in our series carried a high preoperative risk with extensive histories of prior cardiac surgery and complex thoracic anatomy. Yet, both patients demonstrated excellent procedural outcomes with successful transplantation and no obvious procedural complications. Their success supports the viability of stepwise sternotomy to safely perform complex reentry.

## 4. Conclusions

Two-stage sternotomy enables versatile solutions to complex thoracic reentry. A thorough review of preoperative imaging to identify high-risk sites for reentry injury is fundamental to this approach.

## Data Availability

The data underlying this article will be shared on reasonable request to the corresponding author.
